# Delayed diagnosis and misdiagnosis of autoimmune glial fibrillary acidic protein astrocytopathy: a dual-center retrospective cohort study in China

**DOI:** 10.3389/fneur.2026.1968815

**Published:** 2026-10-23

**Authors:** Sheng Lin, Yiqian Huang, Chuming Huang, Xiujun Zheng

**Affiliations:** 1 Department of Neurology, Yuebei People's Hospital, Shaoguan, Guangdong, China; 2 Department of Neurology, Shantou Central Hospital, Shantou, Guangdong, China

**Keywords:** cerebrospinal fluid GFAP-IgG, delayed diagnosis, GFAP-astrocytopathy, infectious meningoencephalitis, misdiagnosis

## Abstract

**Objective:**

To analyze the misdiagnosis and delayed diagnosis of patients with GFAP-Astrocytopathy and to identify the core underlying reasons.

**Methods:**

A total of 42 patients diagnosed with GFAP-Astrocytopathy from July 2020 to March 2026 in two tertiary hospitals were retrospectively included. The patients were defined as having delayed diagnosis if the time from the onset of the initial symptom to the diagnosis was >2 weeks. The misdiagnosis was defined as an incorrect initial diagnosis before the final diagnosis that prompted disease-specific treatment or caused clinically meaningful delay (>7 days) before GFAP-Astrocytopathy was considered, unless autoimmune etiologies were documented and GFAP-IgG testing was ordered within 72 h. Based on this, the patients were divided into the delayed diagnosis group (*n* = 27) and the non-delayed diagnosis group (*n* = 15).

**Results:**

Among the 42 patients, 64.29% (27/42) experienced diagnostic delay, and 80.95% (34/42) were misdiagnosed. The most common misdiagnosis was infectious meningoencephalitis, accounting for 23 cases (54.76%). The proportion of patients first diagnosed in the neurology department was 25.93%(7/27) in the delayed diagnosis group and 53.33%(8/15) in the non-delayed diagnosis group, χ^2^ = 3.115, *P* = 0.039. The non-delayed diagnosis group had significantly higher proportions of neck stiffness and impaired consciousness (*P* < 0.05). The proportion of patients presenting with only isolated fever and/or headache without any localizing neurological syndrome at onset was higher in the delayed diagnosis group [59.26% (16/27) vs. 33.33% (5/15); Fisher's exact test, two-sided *P* = 0.124], but the difference was not statistically significant between the groups. At the time of initial medical consultation, the absence of any localizing neurological syndrome (meningeal, encephalic, myelitic, autonomic, or optic) remained significantly more frequent in the delayed diagnosis group [59.26% (16/27)] compared with the non-delayed diagnosis group [13.33% (2/15), Fisher's exact test, two-sided *P* = 0.004].

**Conclusion:**

Patients with GFAP-Astrocytopathy have a high rate of delayed diagnosis and are frequently misdiagnosed, most commonly with infectious meningoencephalitis. Initial presentation to a non-neurology department was a key factor contributing to diagnostic delay. The absence of localizing neurological syndromes at onset and initial consultation, delayed GFAP-IgG testing, overlapping CSF inflammatory profiles with infectious etiologies, and absence of perivascular radial enhancement on early brain MRI were significantly associated with delayed diagnosis. Concurrent GFAP-IgG testing at first lumbar puncture was associated with a shorter time to diagnosis, though causality cannot be inferred from this observational association.

## 1 Introduction

In 2016, GFAP-Astrocytopathy was formally established as a distinct autoimmune central nervous system disorder (1, 2). GFAP-Astrocytopathy presents with a wide variety of symptoms. The most common initial symptoms include fever, headache, consciousness disorders, limb tremors, ataxia, urinary and fecal dysfunction, mental and behavioral abnormalities, epileptic seizures, and even apathy (3–5). Brain MRI typically reveals linear radial enhancement along the vascular pathways of the cerebral white matter, and spinal imaging demonstrates long-segment cord lesions-these constitute the classic radiological hallmarks (6). The onset of GFAP-Astrocytopathy often manifests with fever, headache, and meningeal irritation signs, which closely resemble viral or tuberculous meningitis. This often leads clinicians to mistakenly prioritize infectious diseases (7). Early antibody testing for GFAP-Astrocytopathy may yield negative results. A small case series reported that 83.3% of patients had negative early antibody results, with subsequent re-examination yielding positive results (8).

In China, the misdiagnosis and delayed diagnosis of GFAP-Astrocytopathy are particularly prominent. Owing to limited awareness of this disease in primary and general hospitals, many patients are initially misdiagnosed with tuberculous meningitis, autoimmune encephalitis, or intracranial space-occupying lesions (9, 10). For example, a case study reported a 24-year-old woman who first presented with fever, headache, and vomiting. MRI showed extensive meningeal enhancement. The patient was initially treated for tuberculous meningitis. However, as her symptoms progressed to urinary retention and tremors, GFAP antibody testing was eventually performed, confirming the diagnosis (11). This delay not only postponed effective treatment but also exposed the patient to unnecessary anti-tuberculosis medications and a prolonged hospital stay.

To date, quantitative research on diagnostic delay in GFAP-Astrocytopathy remains scarce both domestically and internationally. The existing literature consists predominantly of case reports and descriptive summaries of clinical characteristics, lacking systematic analyses of diagnostic delay intervals. In current clinical practice, pathogen screening is the first step when encountering patients with suspected central nervous system infections. Autoimmune etiologies and antibody testing are considered only after pathogen tests return negative and anti-infective treatment has proven ineffective. However, whether this exclusionary diagnostic strategy is appropriate for GFAP-Astrocytopathy remains unclear. If GFAP antibodies were tested simultaneously during the first lumbar puncture in patients presenting with“fever + headache + encephalitis/myelitis” features, could the time to diagnosis be shortened from weeks to within days? This question constitutes the core objective of the present study.

To this end, this study retrospectively analyzes data from 42 patients diagnosed with GFAP-Astrocytopathy at two large tertiary hospitals in China between July 2020 and March 2026. We quantified the diagnostic delay and misdiagnosis rates and identified key factors associated with delayed recognition. These findings may inform future hypothesis-driven studies aimed at optimizing early diagnostic pathways, although the exploratory nature of this analysis precludes immediate algorithmic implementation.

## 2 Methods

### 2.1 Study subjects

Between July 2020 and March 2026, all patients with suspected autoimmune central nervous system (CNS) disorders at two tertiary hospitals (Hospital A: *n* = 28; Hospital B: *n* = 14) were consecutively screened for GFAP-Astrocytopathy. Of 58 CSF GFAP-IgG-positive patients, 16 were excluded (8 with incomplete data, 5 with alternative confirmed diagnoses, 3 with insufficient clinical manifestations), leaving 42 for final analysis (Figure 1). Diagnostic protocols and GFAP-IgG assays remained consistent throughout the study period, with no significant changes in clinical practice or laboratory methods. The ethics committees at both participating centers granted approval for this study. As this study is retrospective and the data have been anonymized, informed consent was waived.

**Figure 1:**
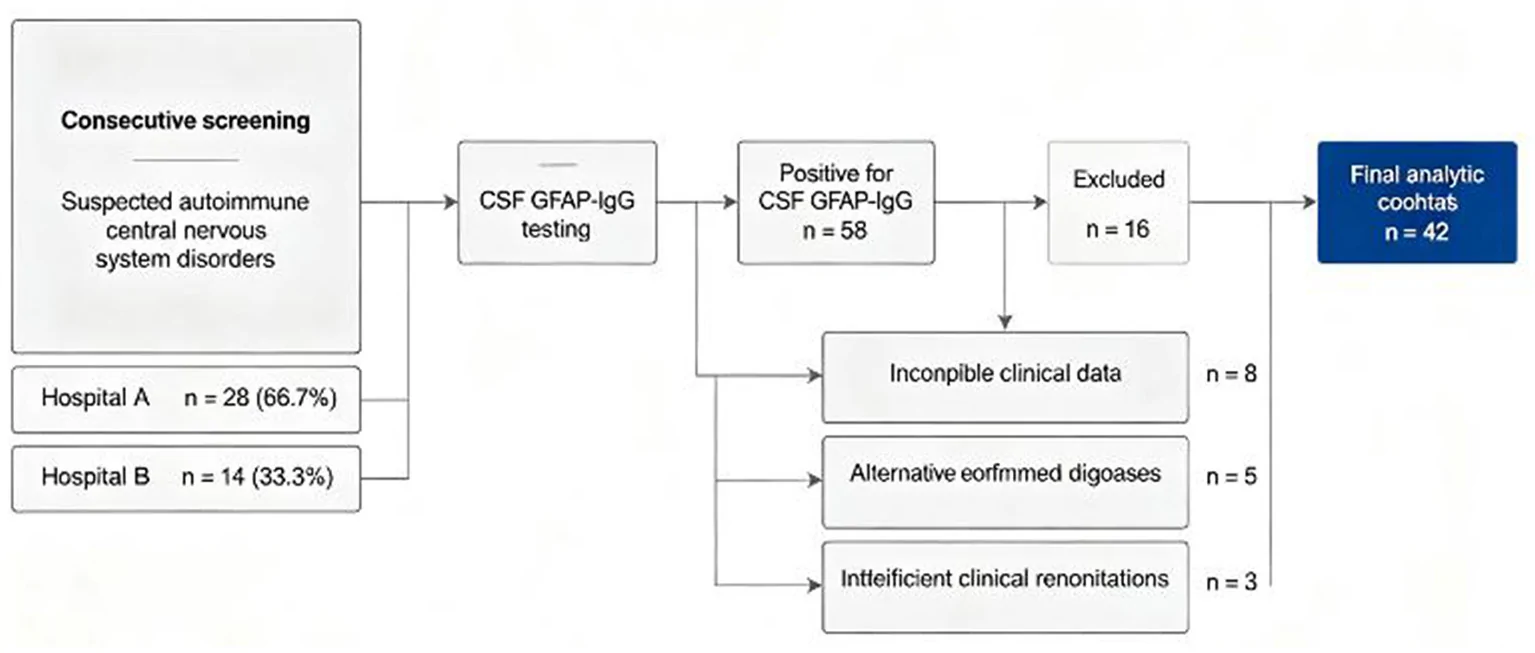
Patient selection flowchart. A total of 58 patients with positive CSF GFAP-IgG were screened. After excluding 16 patients (8 with incomplete data, 5 with alternative confirmed diagnoses, 3 with insufficient clinical manifestations), 42 patients were included in the final analysis.

This study was reported in accordance with the STROBE Statement.

All CSF GFAP-IgG assays were performed at a single designated reference laboratory using identical reagents, protocols, and lot numbers throughout the study period to ensure methodological consistency. Inter-assay variability was monitored using internal controls and remained within acceptable limits.

### 2.2 Inclusion and exclusion criteria

Inclusion criteria: (1) CBA detects positive GFAP-IgG in cerebrospinal fluid. (2) The following clinical manifestations must include at least one of the following: meningitis, encephalitis, myelitis, optic neuritis. (3) Other diagnoses were systematically excluded through comprehensive evaluations: infectious etiologies (CSF Gram stain, culture, multiplex PCR, acid-fast bacilli, TB-PCR, fungal tests); autoimmune encephalitis (anti-NMDAR, LGI1, CASPR2, GABABR, AMPAR); demyelinating disorders (AQP4-IgG, MOG-IgG); paraneoplastic syndromes (anti-Hu, Yo, Ri, CRMP5); and metabolic/toxic/neurodegenerative conditions via biochemical and clinical correlation. All antibody testing employed standardized commercial cell-based assays in the same reference laboratory. (4) Availability of complete clinical data, including demographic information, initial symptom records, first-visit department, complete diagnostic timeline, CSF results, and neuroimaging at initial presentation and during follow-up. Cases with missing data that precluded determination of diagnostic timeline or misdiagnosis status were excluded, with reasons documented to minimize selection bias.

Exclusion criteria: presence of intracranial tumors, central nervous system infections, neurodegenerative disorders, metabolic encephalopathy, toxic encephalopathy, or other types of autoimmune encephalitis.

For patients with visual impairment, optic neuritis was diagnosed based on fundoscopy, MR imaging of the optic nerve, visual field defects, or visual evoked potential abnormalities. All such patients underwent serum AQP4-IgG and MOG-IgG testing to exclude NMOSD and MOGAD. Those with isolated optic neuritis and positive AQP4-IgG or MOG-IgG were excluded from the primary GFAP-Astrocytopathy cohort, unless overlapping syndromes were confirmed by comprehensive clinical and laboratory assessment.

### 2.3 Methods

Access the hospital's electronic medical record database, select the patients that meet the requirements, and retrospectively collect their clinical data, covering demographic characteristics, initial symptoms and their evolution process, the first-visit department and referral path, the timeline of diagnosis, misdiagnosis records, and treatment responses. Laboratory data included cerebrospinal fluid routine biochemistry, pathogen detection, and cerebrospinal fluid GFAP-IgG antibody test results. Imaging data were obtained using 1.5T or 3T MRI scanners for cranial and spinal cord plain and contrast-enhanced scans, with particular emphasis on recording features such as perivascular radial enhancement, dural enhancement, and spinal cord lesion segments. Two neuroradiologists independently and blindly reviewed the images, and reached consensus when opinions differed. Inter-rater consistency was evaluated using the Kappa test, and the results indicated good agreement.

CSF GFAP-IgG was detected using a commercial cell-based assay (Euroimmun, Germany) targeting full-length human GFAP-α, GFAP-**ε**, performed in a single designated reference laboratory strictly in accordance with the manufacturer's protocols. Batch-to-batch quality controls were applied throughout the study period to ensure inter-assay consistency. CSF samples were tested at a 1:1 dilution without titration, as this approach has been widely adopted in routine clinical practice for the detection of central nervous system autoantibodies and offers sufficient diagnostic sensitivity for the purposes of this clinical study. Positivity required unequivocal immunofluorescence, as independently scored by two blinded technicians. Indeterminate results were repeated; if still equivocal, cases were discussed in a multidisciplinary setting and, when feasible, confirmed by tissue-based assay on mouse brain. All positive results were validated by a second independent CBA run to confirm reproducibility. This standardized testing protocol, with rigorous quality assurance measures, ensured methodological consistency across all samples and provided reliable laboratory support for the clinical analyses in this study.

To enable clinically meaningful phenotyping, patients' manifestations were classified into five mutually non-exclusive syndrome categories based on anatomical domains: (1) meningeal (fever, headache, neck stiffness); (2) encephalic (altered consciousness, seizures, ataxia, behavioral changes, limb tremors); (3) myelitic (limb weakness/numbness); (4) autonomic (urinary retention, abdominal distension, dysmotility); and (5) optic (visual impairment with objective confirmation). Isolated fever or headache without other neurological signs was recorded as prodromal features, not assigned to any syndrome category. For diagnostic delay analysis, patients were defined as having “no localizing neurological syndrome at presentation” if they exhibited only isolated fever and/or headache without any meningeal, encephalic, myelitic, autonomic, or optic features-a definition applied identically at initial diagnosis.

Delayed diagnosis was prespecified as >14 days from symptom onset to final diagnosis, a threshold previously used in autoimmune encephalitis studies (12), but not specifically validated for GFAP-Astrocytopathy. This threshold reflects the time by which most infectious etiologies show improvement or are confirmed, beyond which autoimmune causes warrant reconsideration. This threshold was chosen for descriptive comparison between groups and should not be interpreted as a clinically validated cutoff for intervention. Beyond this binary classification, diagnostic timeline components were analyzed continuously: onset-to-first-consultation, onset-to-neurology-referral, onset-to-GFAP-IgG-request, GFAP-IgG-request-to-result, and onset-to-final-diagnosis.

Misdiagnosis was defined as a diagnostic error that prompted disease-specific treatment or caused clinically meaningful delay (>7 days) before GFAP-Astrocytopathy was considered. A working hypothesis of “suspected infectious meningoencephalitis” was not counted as misdiagnosis if the treating physician explicitly documented autoimmune etiologies in the differential and ordered or planned GFAP-IgG testing within 72 h of admission. All suspected misdiagnoses were adjudicated by an independent panel of two neurologists and one infectious disease specialist, who reviewed complete records to determine: (a) whether the initial diagnosis was rendered as definitive, (b) whether specific treatment was administered based on that diagnosis, and (c) whether the incorrect diagnosis led to measurable delay (>7 days) in GFAP-IgG testing or neurology consultation.

The sample sizes of the two groups were not balanced, reflecting the common clinical phenomenon of diagnostic delay in this disease. Given the limited statistical power, no correction for multiple comparisons was performed. The conclusions of this study require verification in a larger sample.

### 2.4 Statistical methods

Data were analyzed with SPSS 25.0. Continuous variables following a normal distribution (assessed by the Shapiro-Wilk test) were presented as means ± standard deviations and compared using the independent-samples *t*-test. For skewed data, medians with interquartile ranges (IQR) were reported and compared using the Mann-Whitney U test. Categorical variables were presented as counts and percentages. Categorical variables were compared using the χ^2^ test when all expected cell counts were at least 5; when any expected cell count was less than 5, Fisher's exact test was used. For 2 × 2 tables analyzed by Fisher's exact test, two-sided exact *P* values are reported. Binary logistic regression was employed to identify independent factors associated with delayed diagnosis, with variables selected based on clinical relevance and univariable screening (*P* < 0.10). To avoid overfitting given the limited number of events (*n* = 27 in the delayed group), no more than three covariates were entered into the final multivariable model. All tests were two-sided, and *P* < 0.05 was considered statistically significant. All reported test statistics and *P*-values were recalculated and verified using the appropriate tests as specified. Additionally, diagnostic timeline components were analyzed as continuous variables using Mann-Whitney U test due to skewed distribution, and their associations with delayed diagnosis were further explored.

## 3 Results

### 3.1 General information

Of the 42 included patients, 28 (66.7%) were from Hospital A and 14 (33.3%) were from Hospital B. No significant inter-center differences were observed in baseline characteristics or diagnostic delay rates (*P* = 0.482).

As shown in Table 1, the two groups comprised 27 and 15 patients, respectively. The proportion of males was higher than that of females in both groups (20/7 vs. 11/4). The mean age at onset was 45.86 ± 16.79 years in the delayed diagnosis group and 48.73 ± 16.41 years in the non-delayed diagnosis group (*t* = −0.540, *P* = 0.592). The proportion of the first diagnosis in the neurology department was 25.93%(7/27) in the delayed diagnosis group and 53.33%(8/15) in the non-delayed diagnosis group. The proportion of patients first diagnosed in the neurology department was significantly higher in the non-delayed diagnosis group than in the delayed diagnosis group (53.33% vs. 25.93%, χ^2^ = 3.115, *P* = 0.039). The initial misdiagnosis rate was 81.48% (22/27) in the delayed diagnosis group and 80.00% (12/15) in the non-delayed diagnosis group (χ^2^ = 0.013, *P* = 0.909).

**Table 1:** Comparison of general data between the two groups of patients.

General data	Delayed diagnosis group (*n* = 27)	Non-delayed diagnosis group (*n* = 15)	t/χ^2^	*P*
Gender (Male/Female)	20/7	11/4	0.014	0.905
Age of onset ($\bar{x}$ ± *s*, years)	45.86 ± 16.79	48.73 ± 16.41	−0.540	0.592
First visit to neurology department [*n* (%)]	7(25.93)	8(53.33)	3.115	0.039
Initial misdiagnosis rate[*n* (%)]	22(81.48)	12(80.00)	0.013	0.909

This table compares the differences between the delayed diagnosis group and the non-delayed diagnosis group in terms of gender, age at onset, proportion of first visit to neurology department, and initial diagnosis misdiagnosis rate.

Among the 34 patients with misdiagnosis, there were 25 males and 9 females. The age at onset was (46.54 ± 16.32) years old. Thirteen cases (38.24%) were initially diagnosed in the neurology department. The most common misdiagnoses were infectious meningoencephalitis (23 cases, 54.76%), multiple sclerosis (2 cases, 4.8%), acute myelitis (2 cases, 4.8%), subacute combined degeneration of the spinal cord (1 case, 2.4%), community-acquired pneumonia (1 case, 2.4%), cerebral infarction (1 case, 2.4%), posterior circulation ischemia (1 case, 2.4%), Guillain-Barré syndrome (1 case, 2.4%), incomplete intestinal obstruction (1 case, 2.4%), and spinocerebellar ataxia (1 case, 2.4%).

### 3.2 Clinical manifestations

According to Table 2, the non-delayed diagnosis group exhibited significantly higher rates of meningeal signs (neck stiffness: 80.00% vs. 33.33%, χ^2^ = 8.182, *P* = 0.004) and encephalic involvement (impaired consciousness: 60.00% vs. 7.41%, Fisher's exact test, two-sided *P* < 0.001) compared to the delayed diagnosis group. In the delayed diagnosis group, autonomic dysfunction was prominent, with urinary difficulty in 51.85% and abdominal pain/distension in 44.44% of patients, though these differences did not reach statistical significance (*P* > 0.05).

**Table 2:** Comparison of clinical symptom incidence between the two groups.

Syndrome Category	Delayed Diagnosis Group (*n* = 27)	Non-delayed diagnosis group (*n* = 15)	χ^2^	*P*
Meningeal involvement				
Fever	14(51.85)	12(80.00)	3.200	0.074
Headache	11(40.74)	12(80.00)	5.960	0.015
Neck stiffness	9(33.33)	12(80.00)	8.182	0.004
Encephalic involvement				
Impaired consciousness	2(7.41)	9(60.00)	–	< 0.001
Epilepsy	2(7.41)	4(26.67)	2.986	0.084
Psychological behavioral abnormalities	3(11.11)	5(33.33)	3.181	0.075
Ataxia	4(14.81)	5(33.33)	1.936	0.164
Limb tremors	8(29.63)	6(40.00)	0.455	0.500
Myelitic involvement				
Limb numbness	9(33.33)	6(40.00)	0.182	0.670
Limb weakness	10(37.04)	6(40.00)	0.037	0.847
Autonomic dysfunction				
Difficulty in urination	14(51.85)	9(60.00)	0.253	0.615
Abdominal pain and distension	12(44.44)	4(26.67)	1.292	0.256
Optic involvement				
Visual impairment	4(14.81)	4(26.67)	0.861	0.354
Other				
Dizziness	12(44.44)	4(26.67)	1.292	0.256
Vomiting	2(7.41)	6(40.00)	6.446	0.011

This table compares the prevalence of meningeal, encephalic, myelitic, autonomic, optic, and other symptoms between the delayed diagnosis group and the non-delayed diagnosis group.

According to Table 3, neck stiffness (40.00% vs. 0.00%, Fisher's exact test, two-sided *P* < 0.001) and impaired consciousness (26.67% vs. 0.00%, Fisher's exact test, two-sided *P* = 0.004) as initial manifestations were significantly more frequent in the non-delayed diagnosis group.

**Table 3:** Comparison of initial presenting syndromes by involvement domain between the two groups [*n* (%)].

Initial symptoms category	Delayed diagnosis group (*n* = 27)	Non-delayed diagnosis group (*n* = 15)	χ^2^	*P*
Meningeal involvement				
Fever	8(29.63)	9(60.00)	3.582	0.058
Headache	8(29.63)	8(53.33)	2.320	0.128
Neck stiffness	0(0.00)	6(40.00)	–	< 0.001
Encephalic involvement				
Impaired consciousness	0(0.00)	4(26.67)	–	0.004
Ataxia	2(7.41)	0(0.00)	1.176	0.278
Epilepsy	1(3.70)	0(0.00)	0.571	0.450
Psychological behavioral abnormalities	1(3.70)	0(0.00)	0.571	0.450
Limb tremors	0(0.00)	1(6.67)	1.852	0.174
Myelitic involvement				
Limb numbness and weakness	9(33.33)	3(20.00)	0.841	0.359
Autonomic dysfunction				
Difficulty in urination	9(33.33)	3(20.00)	0.841	0.359
Abdominal pain and distension	5(18.52)	2(13.33)	0.186	0.666
Optic involvement				
Visual impairment	2(7.41)	0(0.00)	1.176	0.278
Other				
Dizziness	6(22.22)	3(20.00)	0.028	0.867
Vomiting	3(11.11)	1(6.67)	0.221	0.638

This table presents the distribution of initial symptoms across anatomical domains in the delayed diagnosis vs. non-delayed diagnosis groups.

The proportion of patients presenting with only isolated fever and/or headache without any localizing neurological syndrome at onset was higher in the delayed diagnosis group [59.26% (16/27) vs. 33.33% (5/15); Fisher's exact test, two-sided *P* = 0.124], but the difference was not statistically significant between the groups. At the time of initial medical consultation, this absence of localizing neurological syndrome remained more common in the delayed diagnosis group [59.26% (16/27) vs. 13.33% (2/15); Fisher's exact test, two-sided *P* = 0.004]. These findings indicate that GFAP-Astrocytopathy often presents with isolated, non-localizing prodromal symptoms (fever and/or headache without meningeal, encephalic, myelitic, autonomic, or optic signs), which delays clinical recognition and subsequent neurology referral.

As shown in Table 4, the median time from the initial diagnosis to positive CSF antibody results was 16 days in the delayed diagnosis group and 5 days in the non-delayed diagnosis group (*P* = 0.024). The median time from neurology department admission to cerebrospinal fluid antibody positivity was 5 days and 4 days respectively, *P* = 0.130. These results indicate that the pre-specialist delay period-from initial diagnosis to neurology admission-resulting from initial presentation to a non-neurology department is the primary contributor to overall diagnostic delay, rather than differences in diagnostic efficiency after neurology referral.

**Table 4:** Comparison of diagnosis time lines between the two groups (Median Days).

Diagnosis time lines	Delayed Diagnosis Group (*n* = 27)	Non-delayed diagnosis group (*n* = 15)	U	*P*
The time from the initial diagnosis to the positive result of cerebrospinal fluid antibodies	16	5	42.0	0.024
The number of days from the visit to the neurology department until the cerebrospinal fluid antibody test result was positive	5	4	145.0	0.130

This table compares the differences in the time from initial diagnosis to positive cerebrospinal fluid antibody, and the time from referral to neurology department to positive cerebrospinal fluid antibody for the two groups.

### 3.3 Laboratory examination

According to Table 5, the median WBC values for the two groups were 23.00 (8.00, 83.50) × 10^6^/L and 106.00 (20.00, 230.00) × 10^6^/L, Mann-Whitney U test, *P* = 0.126. Regarding the TP level, the median value for the delayed diagnosis group was 0.59 (0.41, 1.17) g/L, and for the non-delayed diagnosis group it was 0.80 (0.43, 1.56) g/L, *P* = 0.465. The median chloride values were 122.87 (119.18, 126.78) mmol/L and 123.20 (114.90, 126.20) mmol/L, *P* = 0.258. The median sugar values were 3.34 (2.69, 3.99) mmol/L and 3.25 (2.62, 4.10) mmol/L, *P* = 0.375. These results indicate that, regardless of diagnostic delay status, the CSF of patients with GFAP-Astrocytopathy showed inflammatory changes characterized by elevated white blood cell counts, elevated protein levels, and normal or slightly decreased chloride and glucose concentrations. Although the median CSF WBC count in the non-delayed diagnosis group was approximately 4.6-fold higher than that in the delayed diagnosis group, this difference did not reach statistical significance. This lack of statistical distinction may be attributable to the small sample size and skewed data distribution, which reduce statistical power to detect a true difference. Therefore, at the conventional α = 0.05 significance level, routine CSF biochemical profiles cannot reliably distinguish between patients who experience diagnostic delay and those who do not. Consequently, relying solely on these conventional CSF indicators is insufficient to effectively predict or shorten the time to diagnosis, underscoring the critical need for specific antibody detection in suspected cases.

**Table 5:** Comparison of routine cerebrospinal fluid test results between the two groups of patients.

Cerebrospinal fluid test indicators	Delayed diagnosis group (*n* = 27)	Non-delayed diagnosis group (*n* = 15)	U	*P*
WBC( × 10^6^/L)	23.00(8.00, 83.50)	106.00(20.00, 230.00)	128.0	0.126
TP(g/L)	0.59(0.41, 1.17)	0.80(0.43, 1.56)	168.5	0.465
Chloride(mmol/L)	122.87(119.18, 126.78)	123.20(114.90, 126.20)	173.0	0.258
Sugar(mmol/L)	3.34(2.69, 3.99)	3.25(2.62, 4.10)	187.5	0.375

This table compares the WBC, TP, chloride, and sugar levels of the two groups of patients, assessing the correlation between inflammatory indicators and diagnostic delay.

Among the 42 patients, 34 (80.95%) had CSF GFAP-IgG submitted concurrently with the first lumbar puncture for pathogen screening, whereas 8 (19.05%) had GFAP-IgG testing ordered only after initial pathogen results returned negative. The median time from neurology admission to antibody positivity was 4 days (IQR 3-6) in the concurrent-testing group and 10 days (IQR 7-14) in the delayed-testing group (Mann-Whitney U test, *P* = 0.002). However, this comparison is observational and subject to confounding by indication: patients with more atypical presentations or lower initial clinical suspicion were more likely to have GFAP-IgG testing deferred. Additionally, center-specific practices, calendar year of diagnosis, and disease severity at presentation may have influenced the decision to order concurrent testing. Therefore, while this association supports the potential value of early antibody testing, it does not establish causality or demonstrate that routine concurrent testing reduces diagnostic delay in all settings.

### 3.4 Imaging examinations

According to Table 6, he proportion of perivascular radial enhancement was 22.22% in the delayed diagnosis group and 60.00% in the non-delayed diagnosis group (χ^2^ = 6.127, *P* = 0.013). The prevalence of meningeal enhancement was 29.63% in the delayed diagnosis group vs. 53.33% in the non-delayed group, and the prevalence of longitudinally extensive spinal cord lesions was 25.93% vs. 20.00%, with neither difference achieving statistical significance (*P* > 0.05). These results indicate that perivascular radial enhancement was significantly less frequent in the delayed diagnosis group, and its absence on early MRI was associated with a higher risk of diagnostic delay, as further confirmed by multivariate regression analysis.

**Table 6:** Comparison of imaging features between the two groups of patients [n (%)].

Imaging examination indicators	Delayed Diagnosis Group (*n* = 27)	Non-delayed diagnosis group (*n* = 15)	χ^2^	*P*
Enhancement of the perivascular radiating ring	6(22.22)	9(60.00)	6.127	0.013
Meningeal enhancement	8(29.63)	8(53.33)	2.364	0.185
Long spinal cord segments involved	7(25.93)	3(20.00)	0.457	0.722

This table compares the occurrence rates of features such as radial enhancement around blood vessels, meningeal enhancement, and long-segment spinal cord involvement in MRI of the head or spinal cord of the two groups of patients.

### 3.5 Analysis of factors affecting delayed diagnosis

Candidate variables for the multivariable model were selected based on clinical relevance and univariable screening at a threshold of *P* < 0.10. Due to the limited number of events (27 cases in the delayed diagnosis group) and the small overall sample size (*n* = 42), the final model was restricted to three prespecified clinically relevant covariates to avoid overfitting: (1) first visit to the neurology department, (2) simultaneous submission of GFAP-IgG testing with the initial CSF examination, and (3) presence of perivascular radial enhancement on brain MRI. These variables were selected a priori because they represent modifiable steps in the diagnostic pathway and an early imaging marker that may influence clinical suspicion.

In an exploratory multivariable logistic regression model (Table 7), the presence of perivascular radial enhancement was associated with lower odds of delayed diagnosis (OR = 0.122, 95% CI: 0.022–0.682, *P* = 0.017). First visit to the neurology department (OR = 0.153, 95% CI: 0.032–0.738, *P* = 0.019) and simultaneous submission of GFAP-IgG testing (OR = 0.234, 95% CI: 0.065–0.840, *P* = 0.026) showed similar associations. However, these findings are exploratory and should be interpreted with caution, as the model could not adjust for potential confounders including center effects, calendar year, disease severity, or clinical suspicion. The Hosmer-Lemeshow test (χ^2^ = 2.145, *P* = 0.976) does not validate model calibration given the small sample size; rather, these results should be considered purely descriptive and hypothesis-generating. Notably, the variables ‘first visit to neurology' and ‘simultaneous GFAP-IgG testing' are closely interrelated in the clinical pathway, and the model did not account for this collinearity or for confounding by indication, as GFAP-IgG testing was not randomly assigned. Therefore, we do not interpret these associations as independent or causal risk factors, but rather as signals that warrant further investigation.

**Table 7:** Logistic regression analysis of factors affecting delay in diagnosis of GFAP-Astrocytopathy.

Variable	B	SE	Wald	*P*	OR	95%CI
First visit to neurology department	−1.877	0.802	5.477	0.019	0.153	0.032–0.738
The simultaneous submission of GFAP-IgG	−1.452	0.651	4.974	0.026	0.234	0.065–0.840
Enhancement of the perivascular radiating ring	−2.104	0.878	5.742	0.017	0.122	0.022–0.682
Constant	−0.825	0.612	1.818	0.178	0.438	–

This table analyzed whether factors such as the first diagnosis in the neurology department, simultaneous submission of GFAP-IgG, and absence of radial enhancement around blood vessels were independent influencing factors for the delay in diagnosis. Odds ratios < 1 indicate an inverse association between the presence of the variable and delayed diagnosis. Due to limited events (*n* = 27), only three covariates were entered to avoid overfitting; wide confidence intervals reflect sample-size constraints and mandate cautious interpretation. These findings are exploratory and should be interpreted as hypothesis-generating, not as establishing independent causal risk factors.

Interpretation of these findings requires caution. First, due to the limited sample size, the model could not accommodate additional potential confounders, including center effects, calendar year of diagnosis, disease severity at presentation, or specific clinical phenotypes. Second, the wide confidence intervals reflect the small number of events and mandate conservative interpretation. Third, because of the exploratory nature of this analysis and the risk of overfitting, these results should be considered hypothesis-generating rather than confirmatory. The identified associations do not establish independent risk factors in a causal sense, but rather highlight clinical and radiological features that may facilitate earlier recognition of GFAP-Astrocytopathy in real-world practice. Larger prospective multicenter studies with adjustment for a broader range of confounders are needed to validate these findings.

## 4 Discussion

This dual-center retrospective cohort study of 42 patients with GFAP-Astrocytopathy in China revealed that diagnostic delay and misdiagnosis remain exceedingly common in real-world clinical practice, affecting nearly two-thirds and over four-fifths of patients, respectively. The most frequently encountered diagnostic pitfall was infectious meningoencephalitis, which accounted for more than half of all misdiagnoses. Our findings indicate that the primary driver of diagnostic delay is not the efficiency of neurological care once patients reach specialists, but rather the pre-specialist period-from initial presentation to neurology referral-during which the absence of localizing neurological syndromes, delayed GFAP-IgG antibody testing, and lack of characteristic perivascular radial enhancement on early MRI collectively impede timely recognition. Importantly, the time from neurology admission to antibody positivity was comparable between groups, suggesting that once patients are appropriately referred, diagnostic workup is generally prompt. These observations underscore that improving early recognition outside neurology settings represents the most critical intervention point for reducing diagnostic delay in this condition.

When contextualized within the existing literature, our findings align with and extend previous observations regarding the diagnostic challenges of GFAP-Astrocytopathy. Since the formal recognition of GFAP-Astrocytopathy as a distinct autoimmune entity in 2016, accumulating case series have consistently highlighted its protean clinical presentations and frequent mimicry of infectious and inflammatory disorders (1, 8). The misdiagnosis rate documented in our cohort parallels that reported in larger case series, where patients were frequently mistaken for tuberculous or viral meningitis, particularly at primary care levels (13, 14). The predominance of infectious meningoencephalitis as the leading misdiagnosis is biologically plausible, given that fever, headache, and meningeal irritation-the hallmark presenting features of GFAP-Astrocytopathy-are virtually indistinguishable from those of acute central nervous system infections. Furthermore, the cerebrospinal fluid profile in GFAP-Astrocytopathy, characterized by lymphocytic pleocytosis and elevated protein with preserved glucose and chloride, overlaps extensively with that of viral and tuberculous meningitis, rendering routine CSF biochemistry a poor differentiator (15). The presence of autonomic dysfunction, observed in a substantial proportion of our patients, represents a clinically valuable clue that could help distinguish GFAP-Astrocytopathy from pure infectious meningoencephalitis, although this feature is neither universally present nor pathognomonic. The frequency of autonomic involvement in our cohort is consistent with the prevalence reported in previous studies, where autonomic symptoms were noted in a substantial proportion of patients. Mechanistically, the predominant involvement of autonomic circuits in GFAP-Astrocytopathy may reflect the tropism of the autoimmune attack toward glial fibrillary acidic protein-expressing astrocytes in periventricular and brainstem regions, where autonomic regulatory centers are located. This topographical predilection could explain the high frequency of urinary retention, sphincter dysfunction, and gastrointestinal dysmotility observed in our cohort. This topographical predilection could explain the high frequency of urinary retention and gastrointestinal dysmotility observed in our cohort. Notably, the time from neurology admission to antibody positivity in our study was markedly shorter than the traditional delay typically associated with stepwise diagnostic algorithms that require prior exclusion of infectious etiologies (16, 17). This observation supports the feasibility of expedited diagnosis through early GFAP-IgG testing at the time of first lumbar puncture, a strategy that has been advocated in recent reviews and expert opinions but has not been rigorously evaluated in prospective studies. The imaging findings in our cohort further corroborate the literature: perivascular radial enhancement, while characteristic, was present in only a subset of patients, and its absence on early MRI did not exclude the diagnosis-consistent with reports that up to half of patients may have normal or non-specific findings on initial neuroimaging (18, 19). Meningeal enhancement, another frequently observed feature, has been described in both GFAP-Astrocytopathy and infectious meningitis, further complicating radiographic differentiation (20). This imaging-clinical dissociation underscores the critical importance of antibody testing rather than reliance on radiological features alone for timely diagnosis. Additionally, emerging evidence has documented overlapping syndromes in which GFAP-Astrocytopathy coexists with anti-NMDAR encephalitis or MOGAD, adding further layers of diagnostic complexity (21, 22). Collectively, these observations emphasize that GFAP-Astrocytopathy should be considered in the differential diagnosis of patients presenting with meningoencephalitis of unclear etiology, particularly when autonomic features are present or when anti-infective therapy fails.

To ensure diagnostic consistency, we applied a standardized case definition adapted from Fang et al. and informed by the Chinese expert consensus (8), with final diagnosis established through a two-step confirmatory process: positive CSF GFAP-IgG on repeat cell-based assay, followed by independent adjudication by three senior neuroimmunologists who confirmed clinical, laboratory, and imaging consistency and excluded alternative diagnoses prior to group assignment. An important observation warrants further scrutiny: the median time from neurology admission to CSF GFAP-IgG positivity was comparable between delayed and non-delayed groups. This similarity may reflect heightened diagnostic awareness among neurologists at tertiary centers, who promptly order antibody testing upon suspicion, effectively eliminating the intra-specialty bottleneck. However, this observation may also be influenced by referral bias-patients with more severe or rapidly progressive presentations, who are more likely to have detectable meningeal or encephalic syndromes, tend to be referred earlier and receive more urgent in-hospital workups, including expedited antibody testing. Conversely, patients with indolent, non-localizing, or autonomic-predominant symptoms experience longer pre-referral delays despite similar post-admission testing intervals, a possibility supported by our finding that isolated fever and/or headache without localizing signs was significantly more frequent in the delayed group at initial consultation. Thus, the similar post-neurology time-to-diagnosis does not entirely rule out the influence of disease severity or phenotypic urgency on the overall diagnostic trajectory. Additionally, institutional practices regarding test prioritization-whether GFAP-IgG was submitted stat or as part of batched weekly runs-may have contributed to inter-individual variation. While our data suggest that neurology referral effectively accelerates diagnosis, they also caution against complacency: the 4–5 day interval from admission to antibody result still represents a meaningful window during which empirical therapies are administered and diagnostic uncertainty persists. Strategies to further compress this interval-such as point-of-care or rapid-turnaround antibody assays-warrant future investigation.

Several important limitations must be considered when interpreting our findings. First, the small sample size and limited number of events in the delayed diagnosis group restricted our capacity to perform robust multivariable adjustment. The logistic regression model included only three prespecified covariates to avoid overfitting, and the wide confidence intervals reflect the statistical instability inherent to small-sample analyses. Consequently, the identified associations should be regarded as exploratory and hypothesis-generating rather than confirmatory. Second, the retrospective design introduces inherent risks of information bias and incomplete data capture. Although we systematically reviewed electronic medical records and excluded cases with missing data that precluded determination of diagnostic timelines, the accuracy of recorded symptom onset dates and referral pathways remains subject to recall and documentation limitations. Third, the generalizability of our findings to other geographic regions or healthcare systems is uncertain. Both participating centers are tertiary referral hospitals located in Guangdong Province, China, where clinical awareness of GFAP-Astrocytopathy may differ from that in primary care settings or in regions with different disease epidemiology. The diagnostic pathways and referral patterns observed here may not reflect those in healthcare systems with different organizational structures or resource availability. Fourth, because we did not include a control group of patients with infectious meningoencephalitis, other autoimmune encephalitides, MOGAD, AQP4-NMOSD, neoplastic, or other inflammatory CNS disorders, we are unable to assess the specificity or predictive value of any clinical, laboratory, or imaging feature for distinguishing GFAP-Astrocytopathy from its mimics. This limitation is particularly relevant given that overlapping syndromes have been increasingly recognized, further complicating diagnostic differentiation in the absence of a comparator cohort. Lastly, multiple statistical comparisons were performed without correction for multiplicity, reflecting the exploratory nature of our analysis; this approach may inflate the risk of type I error, and our findings should be interpreted with appropriate caution. We explicitly acknowledge that the association between concurrent GFAP-IgG testing and shorter time-to-diagnosis is observational and subject to confounding by indication, as the decision to order antibodies early was influenced by clinical judgment, center-specific practices, and pretest probability-factors that themselves may correlate with faster diagnosis.

Despite these limitations, our study provides the first quantitative, multi-center characterization of diagnostic delay in GFAP-Astrocytopathy in the Chinese population and identifies modifiable factors along the diagnostic pathway that warrant targeted intervention. The high rates of misdiagnosis and delayed recognition documented here should serve as a call to action for enhanced educational initiatives aimed at non-neurologists who are often the first point of contact for these patients. Specifically, we suggest that for patients presenting with fever and headache accompanied by subtle autonomic symptoms or without clear localization-particularly when empirical anti-infective treatment fails to produce rapid improvement-the diagnosis of GFAP-Astrocytopathy should be considered early, and GFAP-IgG testing should be performed concurrently with the initial lumbar puncture rather than deferred until after infectious etiologies have been exhaustively excluded. Such an approach, if validated in future prospective studies, has the potential to reduce diagnostic delay from weeks to days, thereby enabling earlier initiation of immunomodulatory therapy and potentially improving long-term neurological outcomes. However, we emphasize that this recommendation remains preliminary and requires prospective validation in larger, unselected diagnostic cohorts with appropriate control populations, along with formal cost-effectiveness analyses, before it can be incorporated into routine clinical algorithms. Future research directions should include multicenter prospective cohorts with standardized early antibody testing protocols, as well as studies examining the pathophysiological mechanisms underlying the prominent autonomic dysfunction observed in this condition, which may provide further diagnostic and therapeutic targets.

The authors declare that the research was conducted in the absence of any commercial or financial relationships that could be construed as a potential conflict of interest.

## Data Availability

The original contributions presented in the study are included in the article/supplementary material, further inquiries can be directed to the corresponding author/s.
